# An Aging and Senescence-Related Gene Signature for Prognosis Prediction in Clear Cell Renal Cell Carcinoma

**DOI:** 10.3389/fgene.2022.871088

**Published:** 2022-05-13

**Authors:** Jiaying Li, Chengpeng Gui, Haohua Yao, Chenggong Luo, Hongde Song, Haishan Lin, Quanhui Xu, Xu Chen, Yong Huang, Junhang Luo, Wei Chen

**Affiliations:** ^1^ Department of Urology, First Affiliated Hospital of Sun Yat-sen University, Guangzhou, China; ^2^ Institute of Precision Medicine, The First Affiliated Hospital, Sun Yat-sen University, Guangzhou, China

**Keywords:** aging, senescence, clear cell renal cell carcinoma, The Cancer Genome Atlas, mRNAs, prognostic model

## Abstract

**Background:** Clear cell renal cell carcinoma (ccRCC) is the most common solid lesion in the kidney. This study aims to establish an aging and senescence-related mRNA model for risk assessment and prognosis prediction in ccRCC patients.

**Methods:** ccRCC data were obtained from The Cancer Genome Atlas (TCGA) and International Cancer Genome Consortium (ICGC) datasets. By applying univariate Cox regression, least absolute shrinkage and selection operator (LASSO), and multivariate Cox regression, a new prognostic model based on aging and senescence-related genes (ASRGs) was established. Depending on the prognostic model, high- and low-risk groups were identified for further study. The reliability of the prediction was evaluated in the validation cohort. Pan-cancer analysis was conducted to explore the role of GNRH1 in tumors.

**Results:** A novel prognostic model was established based on eight ASRGs. This model was an independent risk factor and significantly correlated with the prognosis and clinicopathological features of ccRCC patients. The high- and low-risk groups exhibited distinct modes in the principal component analysis and different patterns in immune infiltration. Moreover, the nomogram combining risk score and other clinical factors showed excellent predictive ability, with AUC values for predicting 1-, 3-, and 5-year overall survival in the TCGA cohort equal to 0.88, 0.82, and 0.81, respectively.

**Conclusion:** The model and nomogram based on the eight ASRGs had a significant value for survival prediction and risk assessment for ccRCC patients, providing new insights into the roles of aging and senescence in ccRCC.

## Introduction

Renal cell carcinoma (RCC) is the most common malignant solid tumor within the kidney, with an incidence of 4.4 per 100,000 (Capitanio et al., 2019). It is reported that 30% of patients are metastatic at diagnosis and almost 30% of the remaining patients will progress to metastases during the follow-up (Capitanio et al., 2019). There are three main RCC subtypes, of which the most prevalent one is clear cell renal cell carcinoma (ccRCC) which accounts for about 70% of all RCC patients (Jonasch et al., 2021). Many markers and models have been put forward to assess the risk of ccRCC, providing useful but insufficient value for prognosis prediction in clinical practice (Klatte et al., 2018). Therefore, establishing a new prediction model is crucial to identify high-risk ccRCC patients with poor prognosis.

Cellular senescence is described as a permanent arresting state of the cell cycle, usually occurring in proliferous cells responding to various stresses (Calcinotto et al., 2019). Senescence happens in several physiological and pathological situations including tissue reconstruction, tissue injury, tumorigenesis, and aging (Calcinotto et al., 2019). Historically, cellular senescence has been described as a protective factor in tumorigenesis by inhibiting the uncontrolled proliferation of tumor-prone cells (Sieben et al., 2018). However, studies also indicated that senescent cells within tissues can facilitate the proliferation and invasion of neighboring pre-neoplastic cells (Kuilman et al., 2008; Ruhland et al., 2016; Kim et al., 2017). In general, the effects of cellular senescence on tumor cells are extremely complex, with both beneficial and pernicious roles in tumor formation, tumor recurrence, and therapeutic efficacy (Sieben et al., 2018). Aging is characterized by a gradual functional decline, resulting in progressive deterioration and tissue dysfunction (McHugh and Gil, 2018). It is revealed as a strong prognostic marker for shorter survival among many cancers (de Magalhães, 2013). Recently, the application of aging-related gene signatures as diagnostic and prognostic biomarkers has caught the attention of many cancer researchers (Liu et al., 2021; Xu and Chen, 2021; Yuan et al., 2021; Yue et al., 2021). However, the prognostic roles of aging and senescence-related genes (ASRGs) and their biological mechanisms remain unclear. There has never been an ASRG signature established for predicting ccRCC patients’ survival.

In this study, we obtained mRNA expression data and corresponding clinical information of ccRCC patients from public databases. Then, we constructed a prognostic signature based on eight aging and senescence-related differentially expressed genes (DEGs) in the TCGA cohort and validated the model in the ICGC cohort. We further performed a functional enrichment analysis to explore the underlying mechanisms. Finally, an ASRG-based nomogram was built to predict the overall survival of patients with ccRCC. Our study provided insights into the key role of cellular aging and senescence in ccRCC development.

## Materials and Methods

### Data Acquisition

Two independent ccRCC cohorts were included in the present study. The mRNA expression and clinicopathological data of The Cancer Genome Atlas-Kidney Renal Clear Cell Carcinoma (TCGA-KIRC) were downloaded from the USCS Xena website (https://xena.ucsc.edu/welcome-to-ucsc-xena) up to August 7 2021, including 606 samples (534 tumor samples and 72 normal samples), of which 517 patients had complete clinicopathological and prognostic data. RNA-seq data and clinical information of another 91 ccRCC tumor samples were obtained from the International Cancer Genome Consortium (ICGC) portal (https://dcc.icgc.org/projects/RECA-EU). The data from TCGA and ICGC are available to the public. This study was exempted from the approval of local ethics committees and strictly followed TCGA and ICGC publication guidelines and data access policies. The clinicopathological features of the two cohorts are summarized in Supplementary Table S1.

A total of 667 cellular aging and senescence-related genes were retrieved from the Molecular Signatures Database (http://www.gsea-msigdb.org/) and are presented in Supplementary Table S2.

### Construction of the Aging and Senescence-Related Prognostic Signature

The aging and senescence-related prognostic signature was constructed using data from the TCGA cohort. The differentially expressed aging and senescence-related genes (AS-DEGs) between ccRCC tissues and normal tissues were obtained by using the Limma R package. *p* value < 0.05 and |log2FC|≥1 were defined as a significant difference, including both downregulated and upregulated genes. To create the AS-DEGs prognostic model, we applied univariate Cox regression, LASSO regression, and multivariate Cox regression to establish a calculation formula as follows: Risk score = αgene(a) × gene expression(a) + αgene(b) × gene expression(b) +…+ αgene(n) × gene expression(n), in which α stands for the coefficient value. We calculated risk scores for each patient. Then, the patients were divided into a low-risk group and high-risk group according to the median risk score.

### Evaluation and Validation of the Aging and Senescence-Related Prognostic Signature

To evaluate and validate the aging and senescence-related prognostic signature, we applied the same calculation formula to each patient in the TCGA and ICGC RECA-EU cohorts. The survminer R package was used to plot the Kaplan–Meier curve to demonstrate the difference in overall survival (OS), disease-specific survival (DSS), and progress-free survival (PFS) between the low- and high-risk groups. A receiver operating characteristic (ROC) analysis was performed to evaluate the sensitivity and specificity of the prognosis prediction. The area under the ROC curve (AUC) was also calculated to quantificate the prediction accuracy. The stats and Rtsne R package were used to explore the distribution difference between the risk groups by the principal component analysis (PCA) and t-SNE, two unsupervised data dimension reduction methods. To examine whether the prognostic signature can be applied as an independent prognostic factor in ccRCC, univariate and multivariate Cox regression analyses were used. We used a heat map to show the correlation between the risk score and other clinicopathological features. A nonparametric test and violin plots were applied to evaluate the relationship between risk scores and clinicopathological features.

### Gene Set Enrichment Analysis

For the Gene Set Enrichment Analysis (GSEA), the GSEA software (version 3.0) was obtained from GSEA website (http://software.broadinstitute.org/gsea/index.jsp). We downloaded the c2. cp.kegg.v7.4. symbols.gmt sets from the Molecular Signatures Database (http://www.gsea-msigdb.org/gsea/downloads.jsp) to evaluate different pathways and molecular mechanisms between the low- and high-risk groups. Based on gene expression profiles and phenotypic grouping, the minimum gene set was set to 5, the maximum gene set was set to 5,000, 1,000 times of re-sampling, and *p* value less than 0.05 was statistically significant.

### Immune Infiltration Analysis

To quantify the immune infiltration level, the annotated gene set file was downloaded from GSEA website. The infiltration levels of 16 immune cells and 13 immune-related pathways in each ccRCC sample were calculated, and the calculation results were exhibited by box plots. Furthermore, we evaluated the TME of ccRCC by using the “ESTIMATE” package to calculate the immune/stromal/ESTIM score.

### Construction of the Predictive Nomogram

To establish a more accurate predictive model capable of predicting the OS of the ccRCC patients, we constructed a nomogram in TCGA cohort containing the risk score and other clinical factors according to the analysis results of the univariate Cox regression. Calibration plots and AUC in ROC curves were applied to evaluate the predictive effectiveness.

### Pan-Cancer Analysis

We downloaded the standardized pan-cancer dataset from the UCSC website, including TCGA, TARGET, and PANCAN cohorts. We extracted ENSG00000147437 (GNRH1) gene expression data and prognostic data of each sample, screening the sample source as solid tissue normal, primary solid tumor, primary tumor, normal tissue, primary blood-derived cancer-bone marrow, and primary blood-derived cancer-peripheral blood. We eliminated the cancer types with less than three samples and finally obtained the expression data of 34 cancer species.

### Statistical Analysis

Data sorting and analysis were conducted by the R 4.1.0 software. An independent sample *t*-test was used for continuous variables with normal distribution and homogeneity of variance. The Wilcoxon rank-sum test was used for non-normal distribution parameters. The Pearson correlation coefficient test was applied to analyze the correlation. *p* value less than 0.05 was considered significant (*p < 0.05, **p < 0.01, and ***p < 0.001). An aging and senescence-based nomogram was constructed and exhibited using the Sangerbox tools, a free online platform for data analysis (http://www.sangerbox.com/tool).

## Results

### Construction of the Aging and Senescence-Related Prognostic Signature

**FIGURE 1:**
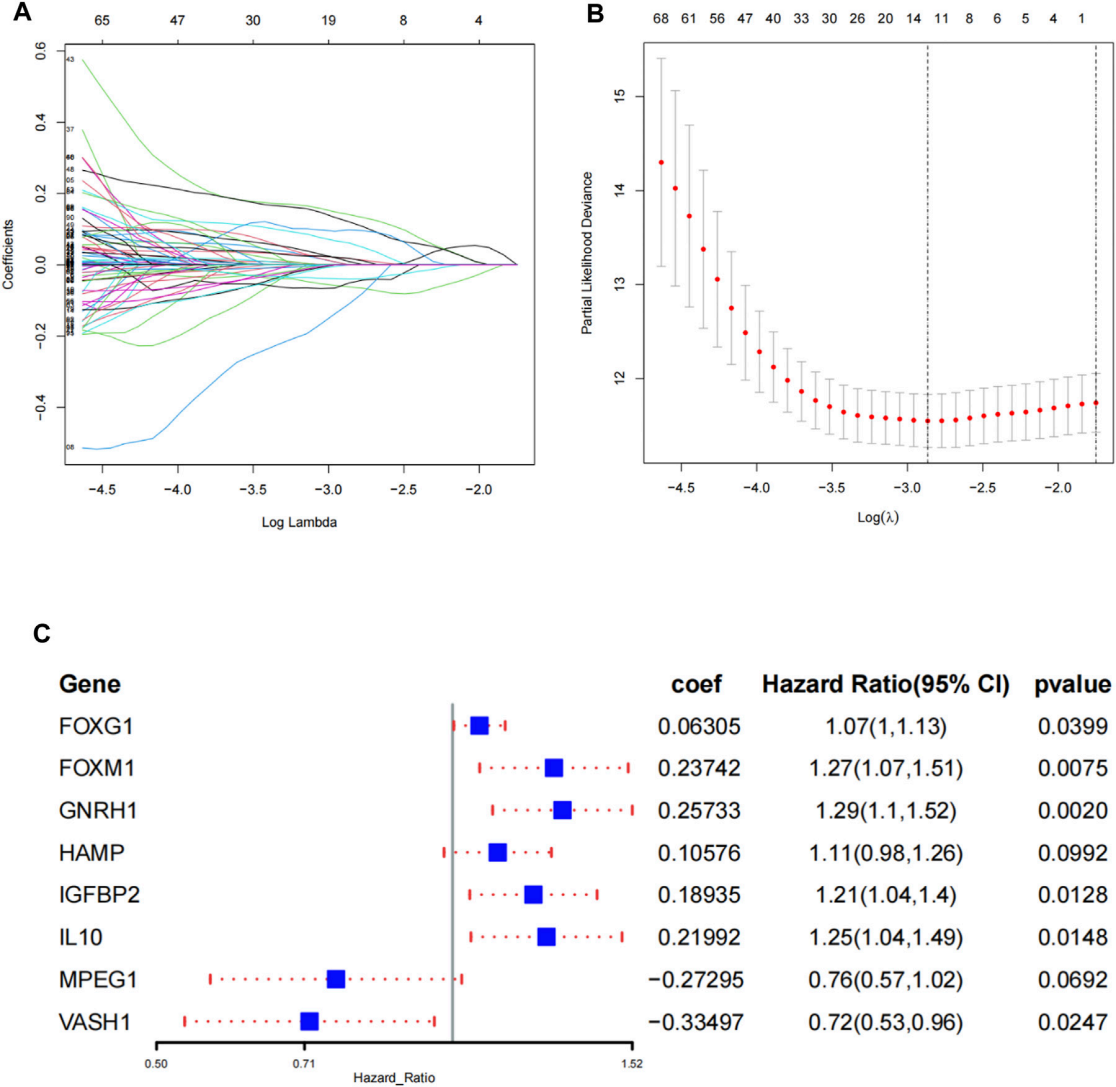
Construction of the prognostic signature in TCGA. **(A)** Select the optimal parameter (lambda) in the least absolute shrinkage and selection operator (LASSO) model. **(B)** LASSO coefficient profiles of the 12 prognosis-associated ASRGs with non-zero coefficients determined by the optimal lambda. **(C)** Identify eight ASRGs to construct a risk signature by the multivariate Cox regression analysis.

Among 667 aging and senescence-related genes derived from the Molecular Signatures Database, 215 genes were differentially expressed (72 underexpressed and 143 overexpressed) between ccRCC tissues and normal tissues in the TCGA cohort (Supplementary Figures S1A,B; Supplementary Table S3). The results of GO and KEGG analyses confirmed that the 215 DEGs were related to aging, cell aging, and cellular senescence (Supplementary Figures S1C,D). Among the 215 DEGs, 59 genes were identified to be associated with OS through the univariate COX regression analysis (Supplementary Table S4). Then, the 59 genes were subjected to a LASSO regression analysis and 12 AS-DEGs were selected (Figures 1A,B). On the basis of the 12 AS-DEGs, a prognostic model was built through a multivariate Cox regression analysis. Eight different AS-DEGs (FOXG1, FOXM1, GNRH1, HAMP, IGFBP2, IL10, MPEG1, and VASH1) were revealed as independent prognostic factors in ccRCC patients (Figure 1C; Supplementary Table S5) and the following formula was generated to calculate the risk score: risk score = (expression of FOXG1 × 0.06305) + (expression of FOXM1 × 0.23742) + (expression of GNRH1× 0.25733) + (expression of HAMP × 0.10576) + (expression of IGFBP2 × 0.18935) + (expression of IL10 × 0.21992) + [expression of MPEG1 × (−0.27295)] + [expression of VASH1 × (−0.33497)].

### Evaluation and Validation of the Aging and Senescence-Related Prognostic Signature

**FIGURE 2:**
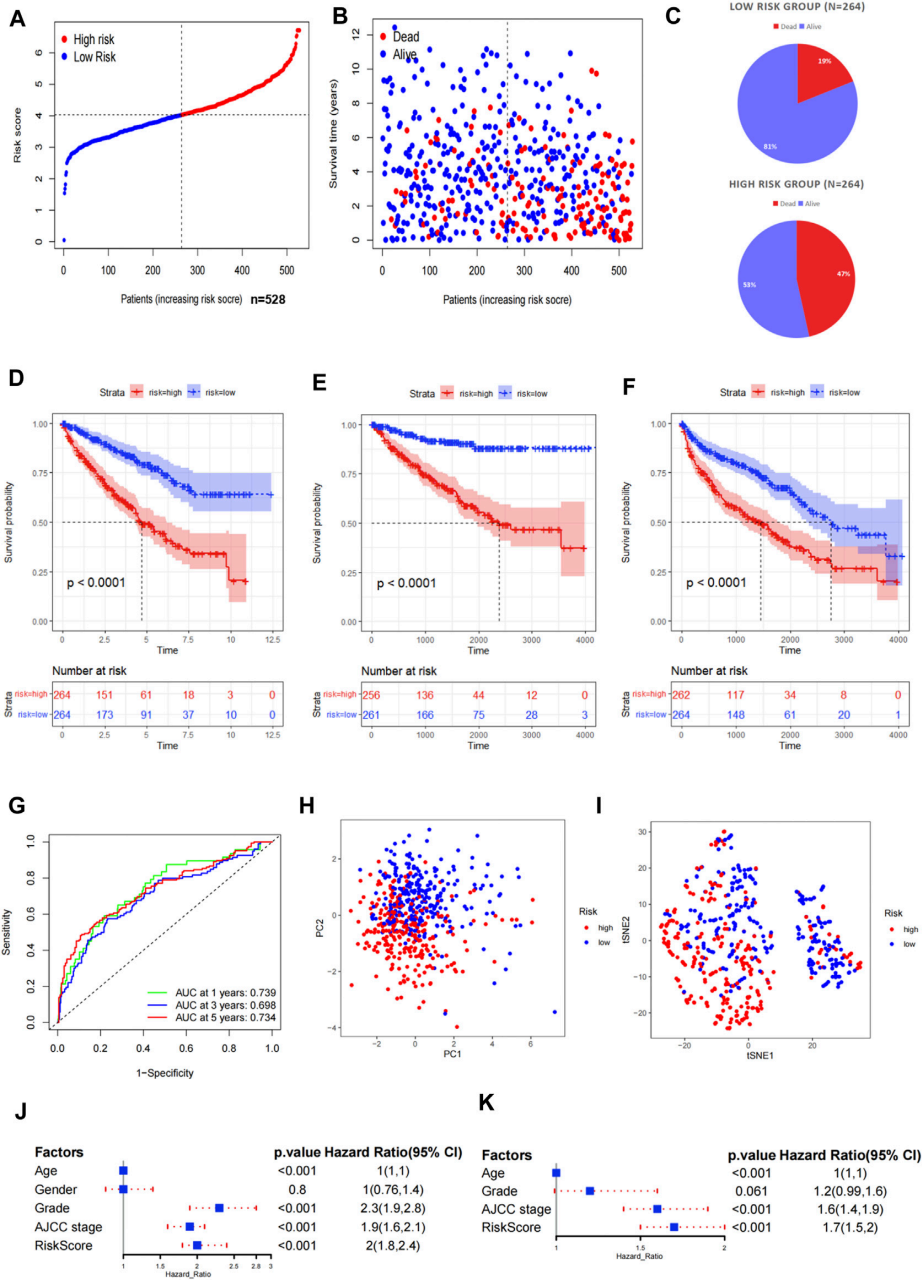
Evaluation of the prognostic signature in TCGA. **(A)** Distribution of the risk scores in the low- and high-risk groups. **(B,C)** Patient distribution in the low- and high-risk groups based on the survival status. **(D–F)** Overall survival (OS), disease-specific survival (DSS), and progress-free survival (PFS) curves stratified by the low- and high-risk groups. **(G)** Time-dependent ROC curves for OS prediction by the ASRG-based signature. **(H,I)** Two risk groups were distinguished by the principal component analysis (PCA) and t-distributed stochastic neighbor embedding (t-SNE). **(J,K)** Univariate and multivariate Cox regression analyses of the signature and other clinical factors.

We calculated the risk score according to the aforementioned calculation formula for each patient in the TCGA cohort and then divided the patients into high- and low-risk groups based on the median risk score. Risk survival status plots and pie charts revealed that the risk score had an adverse association with the survival status of patients (Figures 2A–C). The K–M curves indicated that the high-risk group was associated with poorer OS, DSS, and PFS (*p* < 0.001) (Figures 2D–F). The AUC value for 1-, 3-, and 5-year overall survival were 0.739, 0.698, and 0.734, respectively (Figure 2G). The PCA demonstrated that the high- and low-risk groups presented discrete spatial distributions (Figures 2H, I). We performed Cox regression analyses to evaluate whether the risk score can be used as an independent predictor among other clinical factors such as age, gender, pathological grade, and AJCC stage. Results showed that the risk score remained as an independent prognostic predictor for OS both in the univariate and multivariate analyses containing age, grade, and stage (*p* < 0.001) (Figures 2J,K; Supplementary Table S6).

**FIGURE 3:**
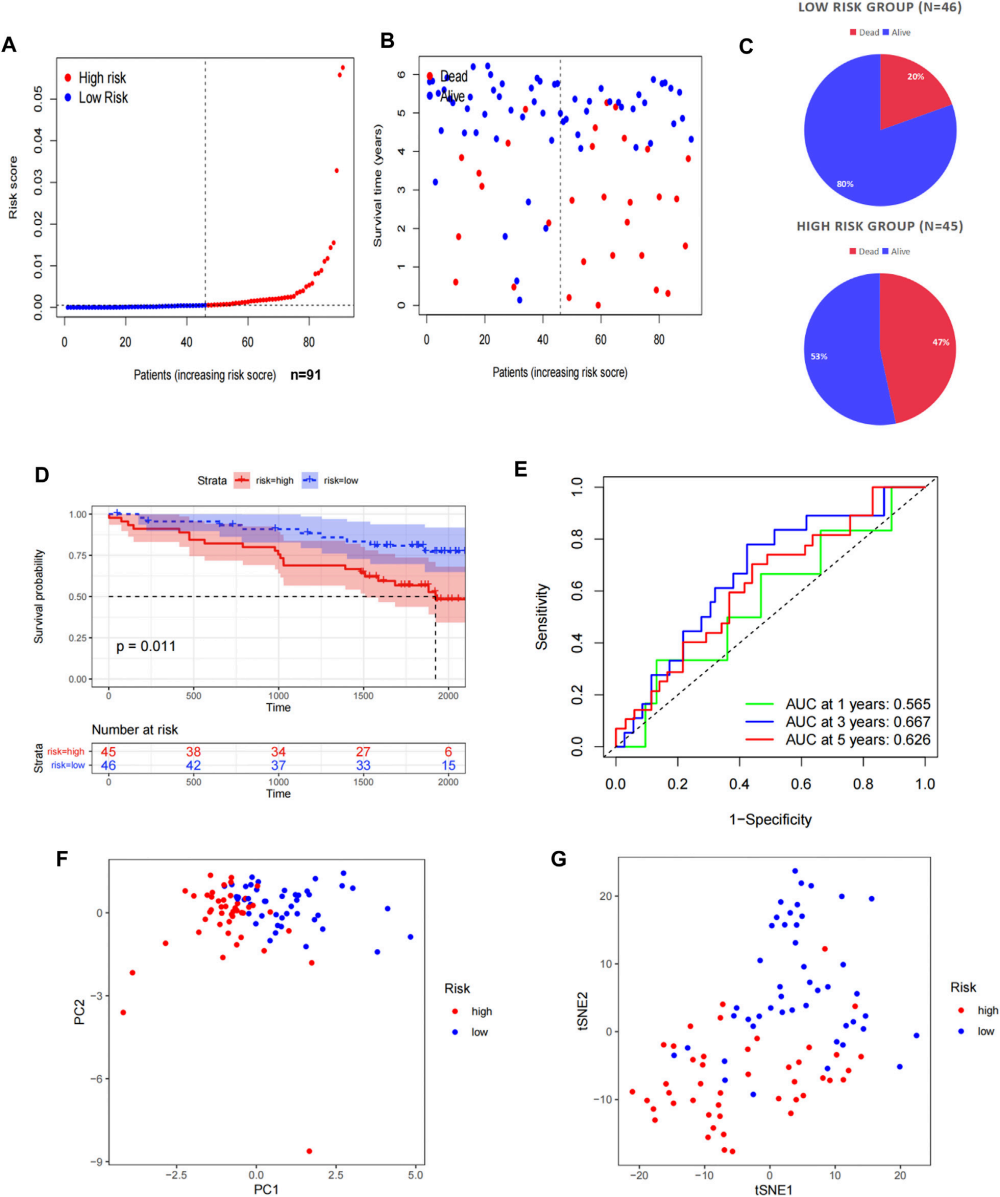
Validation of the prognostic signature in the ICGC. **(A)** Distribution of the risk scores in the low- and high-risk groups. **(B,C)** Patient distribution in the low- and high-risk groups based on the survival status. **(D)** OS curve stratified by the low- and high-risk groups. **(E)** Time-dependent ROC curves for OS prediction by ASRG-based signature. **(F,G)** Two risk groups were distinguished by PCA and t-SNE.

To verify the predictive stability and reliability of our model, we evaluated the patients in the ICGC RECA-EU cohort with the same statistical methods. The verification results were highly in accordance with those in TCGA cohort. The number of deaths increased with increasing risk scores (Figures 3A–C). A survival analysis confirmed that patients in the high-risk group had a shorter OS compared with patients in the low-risk group (Figure 3D). The AUC values of the model for OS were 0.565 at one year, 0.667 at three years, and 0.626 at five years (Figure 3E). Similarly, a significant discrete tendency between two risk groups was shown in the PCA two-dimensional plane (Figures 3F,G).

### Correlation Between the Aging and Senescence-Related Prognostic Risk Signature and Clinicopathological Features

**FIGURE 4:**
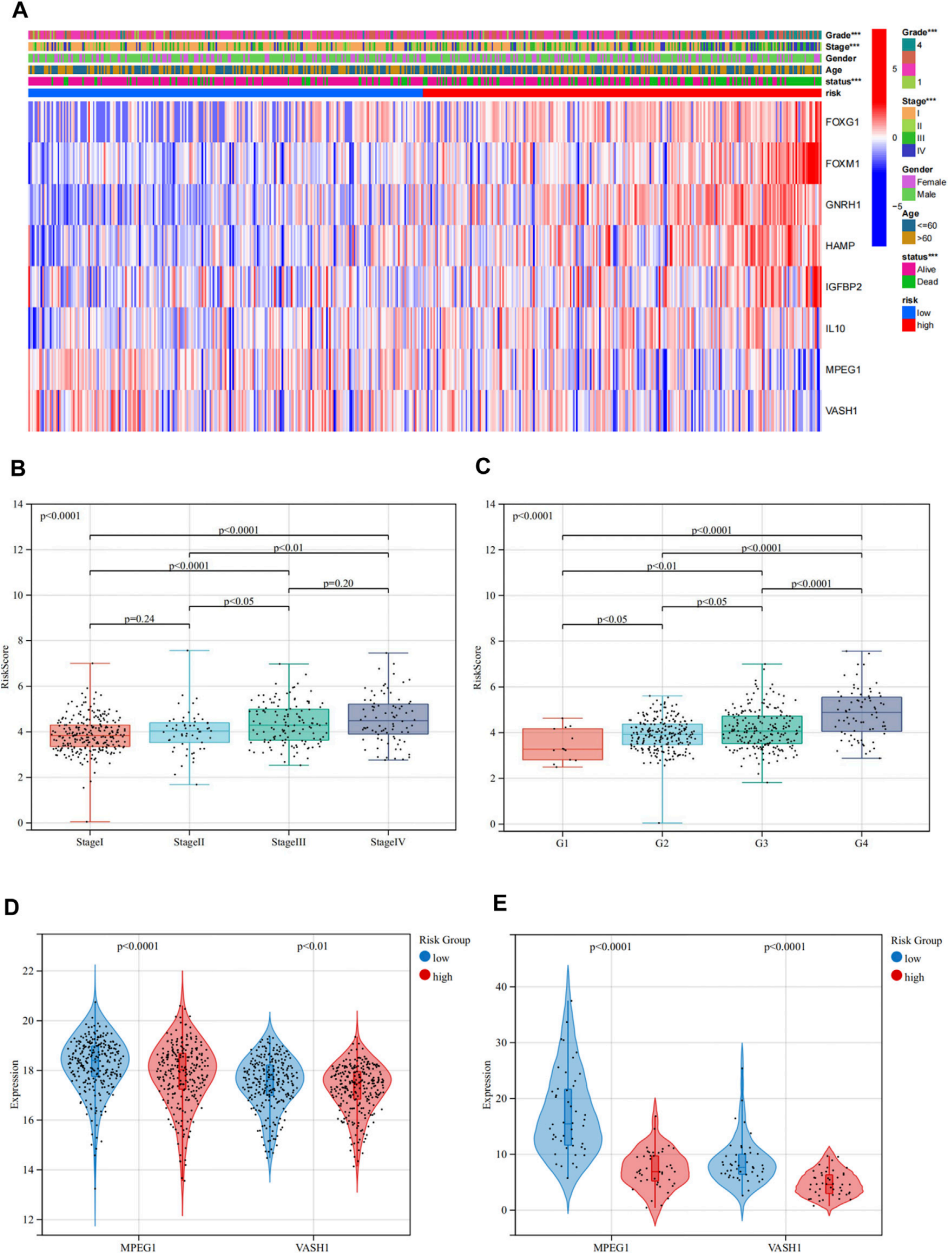
Relationship between the risk groups and clinicopathological features in TCGA. **(A)** Heat map depicting the prognostic signature and clinicopathological features of ASRGs. **(B,C)** Relationship between risk scores and the AJCC stage and pathological grade. **(D,E)** Expression levels of MPEG1 and VASH1 in low- and high-risk groups in TCGA and ICGC cohorts.

Then, we examined the correlation between the risk group and several important clinicopathological features including age, gender, AJCC staging, and pathological grade. The risk group was significantly correlated with the AJCC staging and pathological grade, but not with age and gender. The heat map indicated that patients in the high-risk group were more likely to have a higher AJCC staging and pathological grade than patients in the low-risk group. The gene expression map showed that in the high-risk group, FOXG1, FOXM1, GnRH1, HAMP, IGFBP2, and IL10 were highly expressed, acting as risk factors, while MPEG1 and VASH1 were highly expressed in the low-risk group, as protective factors (Figures 4A,D,E). In the aspect of risk scores, there were significant differences between different stages and grades. Higher AJCC staging and pathological grade were associated with higher risk scores (Figures 4B,C).

### GSEA Analysis Based on Risk Groups

**FIGURE 5:**
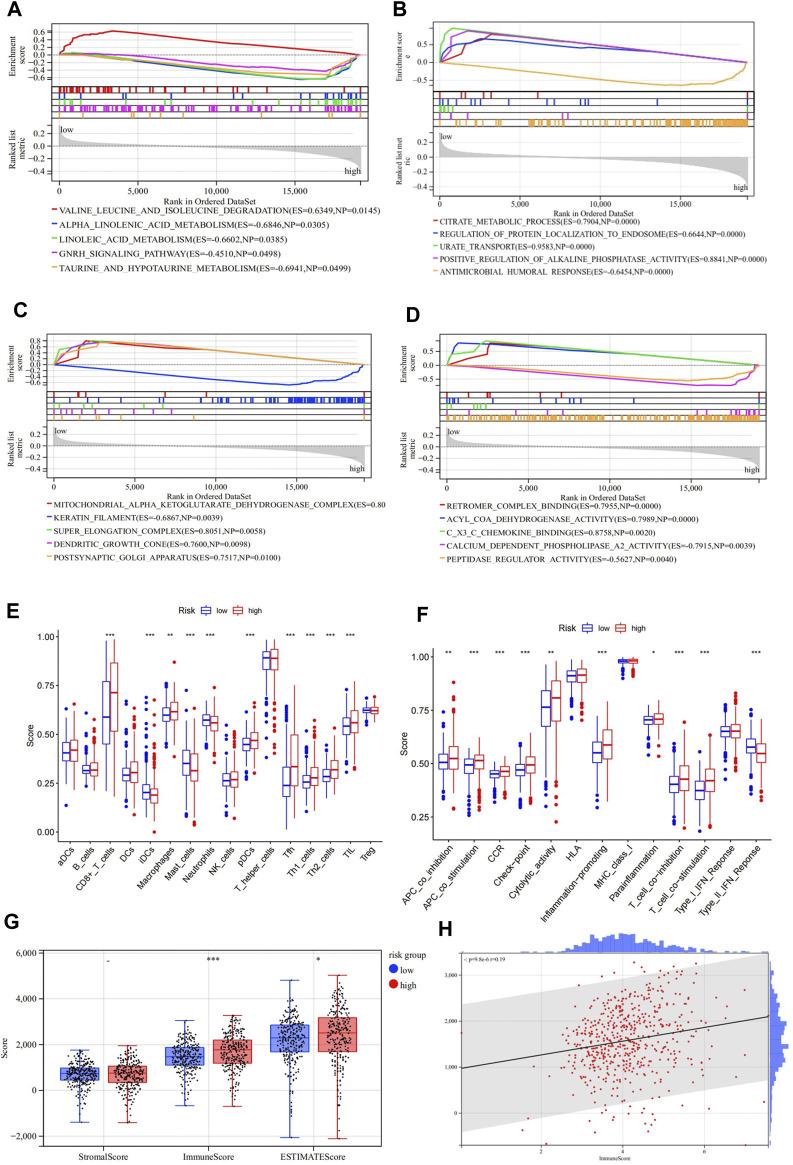
Gene Set Enrichment Analysis (GSEA) of the high-and low-risk groups in TCGA. **(A)** Enriched KEGG pathways between the high- and low-risk groups. **(B–D)** Enriched GOBP, GOCC, and GOMF terms between the high- and low-risk groups. **(E,F)** Enrichment results of the immune cell and immune function scores of the high- and low-risk groups. **(G,H)** Results of the stromal/immune/ESTIMATES scores of the high- and low-risk groups.

We conducted a GSEA analysis based on KEGG and GO pathway enrichment to explore the possible underlying functional mechanisms contributing to different prognoses for patients in the different risk groups in the TCGA cohort. Results of the KEGG terms showed that the low-risk group was significantly enriched in “valine leucine and isoleucine degradation,” while the “alpha-linolenic acid metabolism,” “linoleic acid metabolism,” “GNRH signaling pathway,” and “taurine and hypotaurine metabolism” were significantly enriched in the high-risk group (Figure 5A). For GO terms, the high-risk group was significantly enriched with “antimicrobial humoral response,” “keratin filament,” “calcium-dependent phospholipase A2 activity,” and “peptidase regulator activity” (Figures 5B–D; Supplementary Table S7). These results may shed light on the cellular biological mechanisms of the ASRGs.

### Tumor Immune Infiltration Analysis

We then investigated whether there were differences in the aspect of immune infiltration between the different risk groups. Specifically, we analyzed and compared the infiltration level of 16 immune cells and 13 immune-related pathways. In the present study, most immune cells and immune functions presented significantly discrepant infiltration levels between the low- and high-risk groups (Figures 5E,F; Supplementary Table S8). In particular, CD8^+^ T cells, pDCs, Tfh, TIL, APC co-stimulation, checkpoint, inflammation-promoting, T-cell co-inhibition, T-cell co-stimulation, and type-II IFN response scores were significantly enriched in the high-risk group. Then, the “ESTIMATE” package was applied to evaluate tumor immunity and found similar results. When the risk score increased, the immune scores also increased (Figures 5G,H ; Supplementary Table S9).

### Construction and Evaluation of the Aging and Senescence-Based Nomogram

**FIGURE 6:**
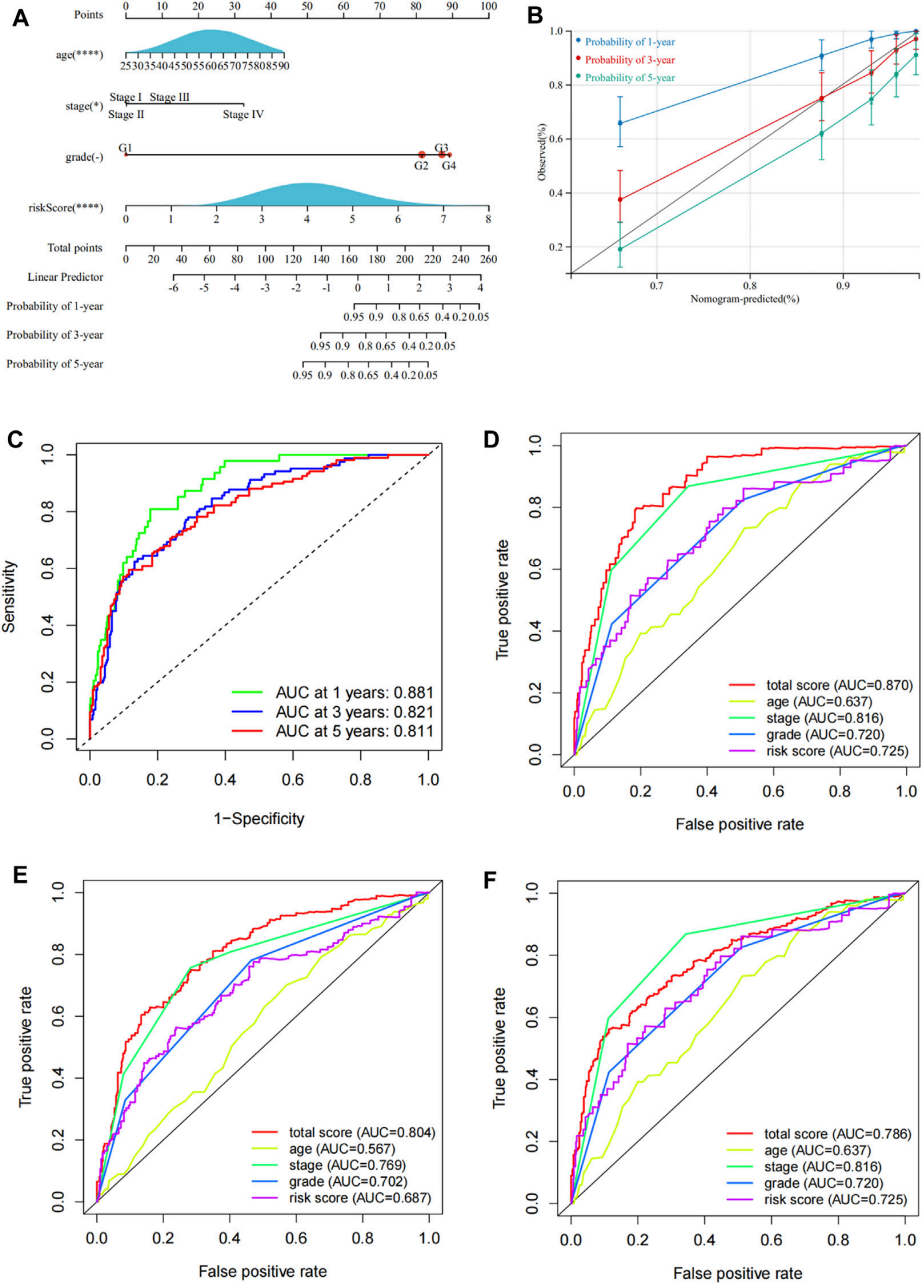
Construction of a nomogram for prognosis prediction. **(A)** Prognostic nomogram including risk scores and other clinical factors. **(B)** Calibration curves of the 1-, 3-, and 5-year OS. **(C)** ROC curves to evaluate the 1-, 3-, and 5-year OS predictive efficiency. **(D–F)** ROC curves to compare the predictive efficiency between the nomogram and other clinical factors for the 1-, 3-, and 5-year OS.

The univariate Cox regression analysis revealed that ASRGs risk score, age, cancer stage, and pathological grade were significant prognostic factors for the overall survival in the TCGA dataset (Figure 2I). Therefore, we used the risk score together with age, stage, and grade to construct a nomogram (Figure 6A). We also draw the calibration curves and ROC to evaluate the predictive accuracy and effectiveness of the prognosis nomogram (Figures 6B,C). For the 1-, 3-, and 5-year OS probability, the AUC of the total score was 0.88, 0.82, and 0.81, respectively (Figure 6C). The ROC curve showed that the total score was more effective than the model constructed only by risk score (Figures 6D–F). The total score outperformed any other clinical factors to predict the 1- and 3-year OS (Figures 6D,E).

### Pan-Cancer Analysis of GNRH1

**FIGURE 7:**
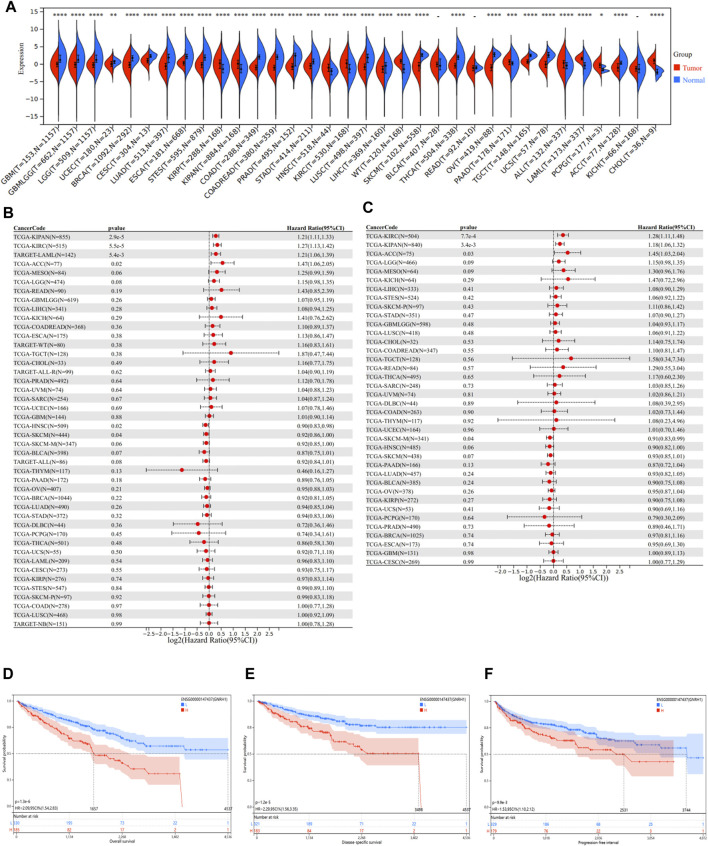
Pan-cancer analysis of GNRH1. **(A)** Expression status of the GNRH1 gene in 34 different cancers and the corresponding normal tissues. **(B)** Correlation between GNRH1 expression and overall survival of different tumors. **(C)** Correlation between GNRH1 expression and disease-specific survival of different tumors. **(D–F)** Kaplan–Meier curves of OS, DSS, and PFS stratified by the low- and high-expression of GNRH1 in TCGA-KIRC.

To investigate the underlying mechanisms relevant to the ASRGs risk model more specifically, we paid attention to the 8 AS-DEGs in the model. Among the six risk factors (FOXG1, FOXM1, GNRH1, HAMP, IGFBP2, and IL10), the coefficients of FOXM1 and GNRH1 were higher than the others, indicating a more prognostically important role. It has been put forward that FOXM1 is a major factor of adverse prognosis in 18,000 cancer cases across 39 human malignancies, confirming the important role of FOXM1 in cancer (Gentles et al., 2015; Gartel, 2017). Therefore, GNRH1 emerged as a focal point of subsequent research. To begin with, we evaluated the disparity of GNRH1 expression between normal and tumor tissues. Data of 34 malignancies showed that GNRH1 was highly expressed in tumor tissues compared with normal tissues in 10 tumors, including KIRC, KIRP (kidney renal papillary cell carcinoma), KIPAN (pan-kidney cohort), LAML (acute myeloid leukemia), HNSC (head and neck squamous cell carcinoma), PAAD (pancreatic adenocarcinoma), and WT (high-risk Wilms tumor) (Figure 7A). On the other hand, in other 21 tumors, GNRH1 was expressed at a lower level in tumor tissues than in normal tissues (Figure 7A).

Given the significant discrepancy in expression levels between tumor and normal tissues in most tumor types, we next investigated the relationship between GNRH1 expression and the prognosis of different tumors. We found that the higher GNRH1 was expressed, the shorter OS was observed in KIPAN (*p* = 2.9e-5), KIRC (*p* = 5.5e-5), LAML (*p* = 5.4e-3), and ACC (adrenocortical carcinoma) (*p* = 0.02). DSS analysis data showed a similar pattern that higher GNRH1 expression was associated with poorer prognosis in KIPAN (*p* = 3.4e-3), KIRC (*p* = 7.7e-4), and ACC (*p* = 0.03). On the contrary, a lower expression of GNRH1 was correlated with shorter OS in HNSC (*p* = 0.02) and SKCM (skin cutaneous melanoma) (*p* = 0.04) (Figures 7B,C). The Kaplan–Meier survival analysis demonstrated that the higher expression of GNRH1 was connected with poorer OS (*p* < 0.001), DSS (*p* < 0.001), and PFS (*p* < 0.001) in ccRCC (Figures 7D–F).

The aforementioned findings revealed that though GNRH1 was differently expressed between tumor and normal tissues in most tumor types, the GNRH1 expression only correlated with the prognosis of a few types of tumors, in particular with RCC. To further explore the role of GNRH1 in cancers, we downloaded the interaction network of the top 20 proteins binding with GNRH1, which was available from the STRING website (Supplementary Figure S2A). The PPI network indicated that GNRH1 interacted with several essential oncogenes and tumor suppressor genes, such as KiSS1 (Kisspeptin), AKT1/3 (serine/threonine kinase 1/3), and MAPK1/3 (mitogen-activated protein kinase 1/3) (Revathidevi and Munirajan, 2019; Ji et al., 2013; Burotto et al., 2014). In addition, the GEPIA2 tool was used to evaluate the expression correlation of the hot tumor-related genes and GNRH1 in ccRCC. We found that the GNRH1 expression level was negatively correlated with MAPK1 and MAPK3 (Supplementary Figures S2B,C). There was evidence indicating that the MAPK/ERK signaling node can function as a tumor suppressor (Burotto et al., 2014). In addition, according to the GNRH1 expression level, patients in the TCGA cohort were divided into a high-expression group (≥50%) and low-expression group (<50%). The GSEA analysis revealed that the MAPK pathway is enriched in the GNRH1 high-expression group (*p* < 0.05), indicating a potential upstream and downstream relationship between the GNRH1 and MAPK pathways (Supplementary Figure S2D). Therefore, we could speculate that GNRH1 negatively affected the prognosis of ccRCC through the MAPK pathway. More importantly, a study confirmed that MAPK pathways mediate GnRH1-stimulated FSHB promoter activities in immortalized murine gonadotrope cells (Wang et al., 2008). However, how GNRH1 interacts with the MAPK pathway in ccRCC still needs more *in vitro* and *in vivo* experiments.

## Discussion

Cellular senescence was first described in 1961 by Hayflick and Moorhead when they found that human diploid fibroblasts *in vitro* could divide to a maximum number before they arrested their growth (Hayflick and Moorhead, 1961). Generally speaking, cellular senescence is considered a stable arresting status of the cell cycle in response to various stresses (Herranz and Gil, 2018). Senescent cells were observed to cumulate in the aging process and play roles in aging-related diseases, such as osteoarthritis and osteoporosis, atherosclerosis, diabetes, glaucoma, and neurodegeneration diseases (van Deursen, 2014; McHugh and Gil, 2018; Calcinotto et al., 2019). Moreover, it was revealed that cellular senescence had complicated effects on tumor by suppressing tumor development at an early stage, promoting tumor growth in the later stage and participating in tumor relapse after chemotherapy (Pérez-Mancera et al., 2014; Calcinotto et al., 2019). Recently, a study detected that various aging/senescence-induced genes (ASIGs) were upregulated in malignant diseases compared with healthy control samples, and an scRNA-seq analysis revealed that all cancer entities (chronic myelogenous leukemia, colorectal cancer, hepatocellular carcinoma, lung cancer, and pancreatic ductal adenocarcinoma) evaluated in the study comprised a cellular subpopulation expressing aging/senescence-associated genes (Saul and Kosinsky, 2021). Xu and Chen (2021) constructed an aging-related gene signature, which were was significantly correlated with the diagnosis and prognosis of lung adenocarcinoma. Yue et al. (2021) also built a novel gene signature associated with aging, which can be used to predict the prognosis of colorectal cancer. Similar research results were also found in breast cancer and ovarian cancer (Liu et al., 2021; Yuan et al., 2021). There are several studies investigating the role of senescence in kidneys. Evidence suggests that cellular senescence is important in the pathogenesis of different forms of renal damage, including acute and chronic kidney disease, and renal transplantation (Li and Lerman, 19792020). A previous study showed that senescence-associated protein p400 is a prognostic biomarker in renal cell carcinoma and lowered expression of p400 associated with worsening prognosis (Macher-Goeppinger et al., 2013). However, no studies have attempted to construct an aging and senescence-related prognostic model of RCC.

A number of studies have attempted to develop models to predict the prognosis in ccRCC patients based on gene sequencing and clinical data (Li et al., 2020a; Chen et al., 2020; Hua et al., 2020; Wang et al., 2020; Zhang et al., 2020; Zhong et al., 2020; Xing et al., 2021). However, few results have been applied in clinical practice. In the present study, we established an aging and senescence-based signature of eight AS-DEGs, including FOXG1, FOXM1, GNRH1, HAMP, IGFBP2, IL10, MPEG1, and VASH1. The ASRG signature was proven to be an independent risk factor for patients with ccRCC and was significantly associated with patients’ prognosis and clinicopathological features. Furthermore, a nomogram based on ASRG signature was established. Our aging and senescence-related prognostic signature showed good performance and AUC values to predict 1-, 3-, and 5-year OS in the training set were 0.739, 0.698, and 0.734, respectively. Moreover, our nomogram combining risk scores and other clinical factors showed superior discrimination and calibration, with AUC values for predicting 1-, 3-, and 5-year OS in the training set reaching 0.88, 0.82, and 0.81, respectively. These results showed that the present approach had an excellent ability to predict the survival in patients with ccRCC.

The aging and senescence-related prognostic risk signature proposed in the present study is composed of eight genes, which are associated with tumor initiation, proliferation, metastasis, and drug resistance. The gonadotropin-releasing hormone (GNRH1) triggers the release of the follicle-stimulating hormone and luteinizing hormone from the pituitary (Canzian et al., 2009). A previous study revealed that IKK-β and NF-κB mediate aging-related hypothalamic GNRH decline, and GNRH treatment amends aging-impaired neurogenesis and decelerates aging (Zhang et al., 2013). GNRH1 was also reported to be correlated with the prognosis of cancer. One study found that GNRH1 expression could be considered a method of tumor cell metastatic spread detection in patients with gynecological malignances (Andrusiewicz et al., 2011). A recent study also found that GNRH1 and the leukotriene B4 receptor (LTB4R) might be novel immune-related prognostic biomarkers in ccRCC (Wu et al., 2021). Our study further explored the role of GNRH1 in different tumors. An expression analysis revealed that GNRH1 was differentially expressed between tumor tissues and the corresponding normal tissues in the majority of tumor types. A survival analysis suggested that increased GNRH1 expression related to poorer prognosis in KIPAN, KIRC, LAML, and ACC. Nevertheless, in HNSC and SKCM, the lower expression of GNRH1 in patients had poorer OS. A PPI network indicated the GNRH1 interacted with several important cancer-related proteins, including AKT1/3, MAPK1/3, and KISS1. A further analysis found that the GNRH1 expression level was negatively correlated with MAPK1 and MAPK3. These results could serve as a basis for future studies on the role of GNRH1 in human malignancies.

Forkhead box protein M1 (FOXM1) is a transcription factor of the Forkhead family that is required for cell proliferation of normal cells (Gartel, 2017). It is closely involved with the processes of cell proliferation, self-renewal, and tumorigenesis (Liao et al., 2018). It is also associated with aging and senescence. In hepatocellular carcinoma, a long non-coding RNA-encoded peptide PINT87aa could induce cellular senescence by blocking FOXM1-mediated PHB2 (Xiang et al., 2021). Another study reveals that FOXM1 induction in elderly and Hutchison-Gilford progeria syndrome fibroblasts prevents aneuploidy and ameliorates cellular aging phenotypes (Macedo et al., 2018). FOXM1 also plays an important role in ccRCC. Experiments showed that the downregulation of FOXM1 reduced the expression and function of the matrix metalloproteinase-2/9 (MMP-2/9) and vascular endothelial growth factor (VEGF), leading to the inhibition of tumor invasion, migration, and angiogenesis (Xue et al., 2012). Another member of the Forkhead family, Forkhead box G1 (FOXG1) was also associated with aging and senescence and is important for a variety of cellular events in cancer cells (Chan et al., 2009; Verginelli et al., 2013; Chen et al., 2018; Wang et al., 2018; Zheng et al., 2019). In glioblastoma, FOXG1 knockdown in brain tumor-initiating cells causes the downregulation of the neural stem/progenitor and increased replicative senescence (Verginelli et al., 2013). In glioma, FOXG1 has been shown to regulate cell proliferation and cell cycles (Chen et al., 2018). In addition, FOXG1 plays a key role in mediating cancer cell metastasis through the Wnt/β-catenin pathway in HCC cells and predicts HCC prognosis after surgery (Zheng et al., 2019). Hepcidin (HAMP) plays a key role in tumor cell proliferation and metastasis (Guo et al., 2015; Bao et al., 2016; Wu et al., 2020). Experiments indicated that maladjusted hepcidin signaling was associated with an increased risk of HCC (Bao et al., 2016). It was also discovered that HAMP promoted lung cancer cell homing and fostered tumor progression (Guo et al., 2015). Insulin-like growth factor-binding protein 2 (IGFBP2) is secreted by white adipocytes and contributes to the prevention of diet-induced obesity and age-related insulin resistance (Li and Picard, 2010). It is also an essential oncogenic protein that has both extracellular and intracellular functions with a clear causal role in cancer development (Chua et al., 2016; Li et al., 2020b; Sun et al., 2021; Wei et al., 2021). Interleukin-10 (IL-10) has been thought to promote tumor immune escape by diminishing anti-tumor immune response in the tumor microenvironment (Mannino et al., 2015). Multiple studies have found a positive correlation between IL-10 levels and poor prognosis in various cancers, including melanoma, lung cancer, and T/NK-cell lymphomas (Boulland et al., 1998; Boyano et al., 2000; Li et al., 2014). Two protective factors (MPEG1 and VASH1) in our prognostic model were mentioned in other studies as well. Macrophage-expressed gene 1 (MPEG1) was downregulated in hepatocellular carcinoma patients with poor prognosis (Zhu et al., 2019). Vasohibin-1 (VASH1) is a negative feedback regulator of angiogenesis (Takahashi et al., 2016). The expression of VASH1 is downregulated during replicative senescence of endothelial cells, which might be a risk of deterioration of vascular homeostasis and age-related vascular diseases (Sato, 2018). In the aspect of cancer, it inhibits the advancement of ovarian cancer by producing various angiogenic factors (Takahashi et al., 2016).

Some of the KEGG/GO pathways shown in Figure 5 have been reported to be related to aging and senescence. For example, the reduction in blood levels of branched-chain amino acids (leucine, valine, and isoleucine) is one of the most consistent aging signatures across human studies (Le Couteur et al., 2020a; Le Couteur et al., 2020b). Pro- and ant-inflammatory bioactive lipids which participate in important cellular processes come from linoleic acid and alpha-linolenic acid through desaturases, and the activity of desaturases decreases with age (Das, 2021). Also, a study reveals that GnRH-I and GnIH are the pivotal neuropeptides regulating the hypothalamic–pituitary–gonadal axis in mammals during aging (Mate et al., 2022). In addition, a study shows that the activities of the mitochondrial citric acid cycle enzymes decrease during the aging process, resulting in secondary effects of citrate accumulation (Yarian and Sohal, 2005).

This study has several limitations. First, the data analyzed was obtained from public databases, therefore, the mRNA and protein expression of the eight genes for constructing the model needs support from experimental evidence. Second, there are many other factors affecting the prognosis of ccRCC patients, such as tumor metabolism, tumor immunity, and non-coding RNA. We still need more data to completely verify the model’s reliability. Finally, the underlying mechanisms of this model remain unknown. In particular, how GNRH1 influences tumor development in ccRCC requires further experiments *in vivo* and *in vitro*.

In summary, we established a novel prognostic model of eight aging and senescence-related genes, which has significant value in predicting ccRCC survival. This signature is expected to be applied as a novel method for identifying high- and low-risk ccRCC populations and help in learning more about the mechanism of aging and senescence in ccRCC.

## Data Availability

The original contributions presented in the study are included in the article/Supplementary Material, further inquiries can be directed to the corresponding authors.

## Supplementary Material

Supplementary Figure S1 Differentially expressed and functional enrichment analysis. **(A)** Heatmap was used to show the expression of all differential genes in tumor and normal tissues. **(B)** Volcano plot was used to show differentially expressed genes (DEGs) in TCGA ccRCC samples. Each point represents an mRNA. Red means a high expression level, blue means low expression level, and p value <0.05 and |log2FC|≥1 are meaningful. FDR stands for false discovery rate. **(C,D)** GO and KEGG analyses of DEGs.

Supplementary Figure S2 Enrichment analysis -related genes. **(A)** Interaction network of 20 GNRH1-binding proteins, which were experimentally determined by the STRING tool. **(B,C)** Expression correlation between GNRH1 and MAPK1, and MAPK3 by the GEPIA2 approach. **(D)** GESA of the MAPK pathway enrichment in the GNRH1 high- and low-expression groups.
