# Supplementary figures and images for: An Aging and Senescence-Related Gene Signature for Prognosis Prediction in Clear Cell Renal Cell Carcinoma
Source: Front Genet. 2022 May 13;13:871088. doi: 10.3389/fgene.2022.871088 (PMC9136295; doi:10.3389/fgene.2022.871088)

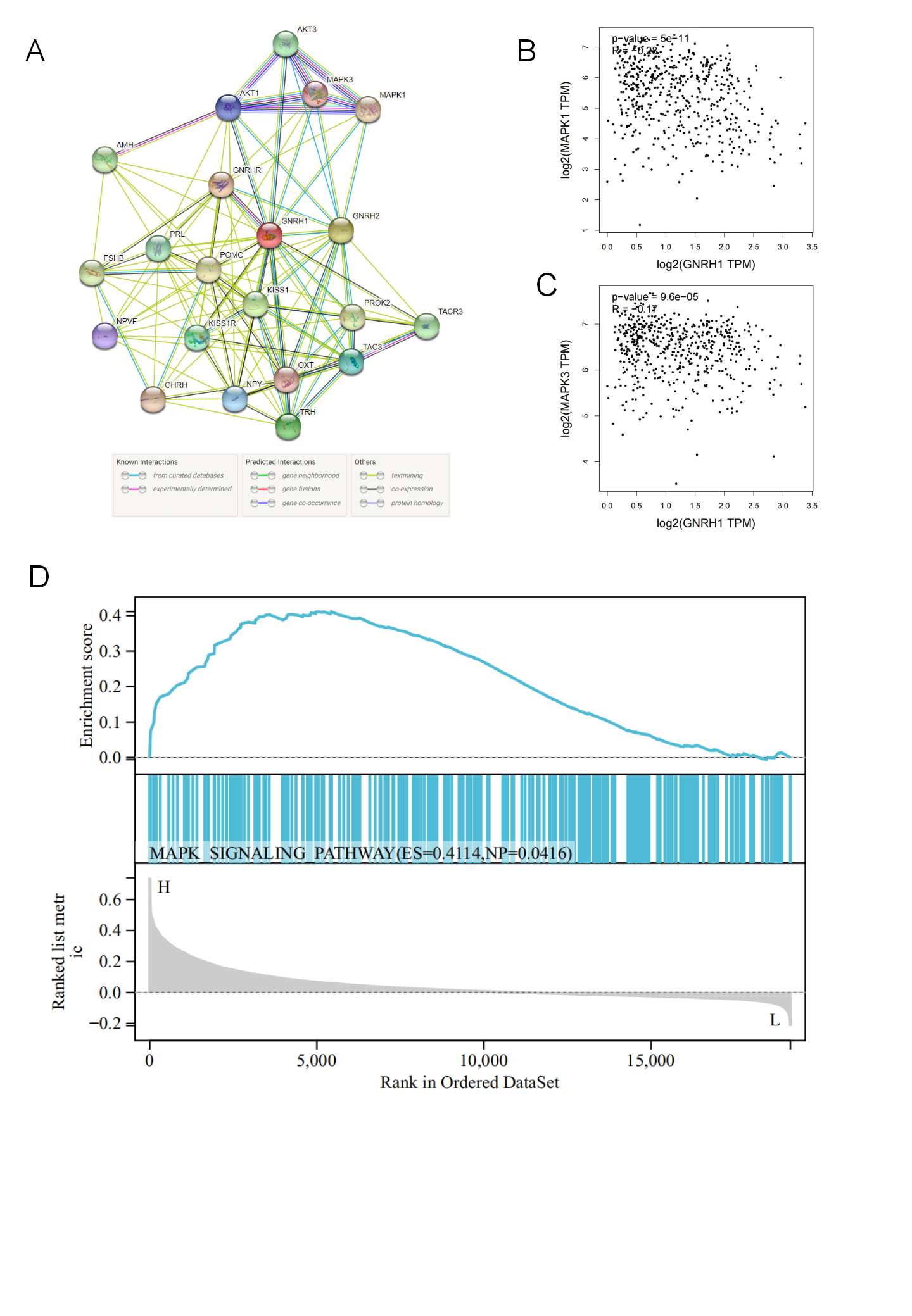

Supplement: Supplementary file 2 [file Image2.TIF]

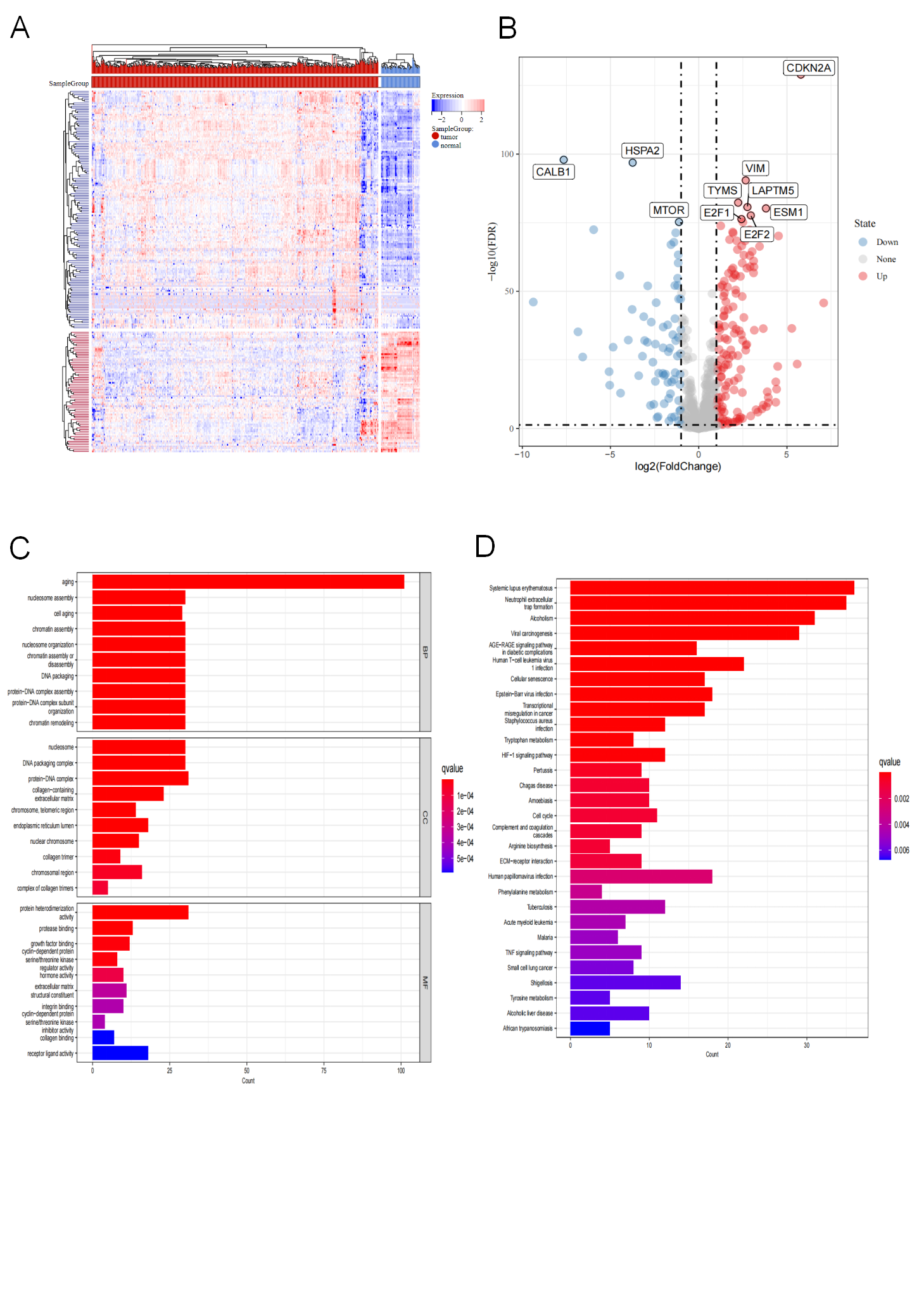

Supplement: Supplementary file 3 [file Image1.TIF]
